# Walking and weakness in children: a narrative review of gait and functional ambulation in paediatric neuromuscular disease

**DOI:** 10.1186/s13047-020-0378-2

**Published:** 2020-03-02

**Authors:** Rachel A. Kennedy, Kate Carroll, Jennifer L. McGinley, Kade L. Paterson

**Affiliations:** 1 Department of Neurology, The Royal Children’s Hospital, Parkville, Vic Australia; 2 Murdoch Children’s Research Institute, Parkville, Vic Australia; 3 Department of Physiotherapy, The University of Melbourne, Parkville, Vic Australia

**Keywords:** Neuromuscular disease, Duchenne muscular dystrophy, Spinal muscular atrophy, Charcot-Marie-tooth disease, Myopathy, Weakness, Paediatric, Gait, Functional ambulation, Assessment

## Abstract

**Background:**

Weakness is the primary impairment in paediatric neuromuscular diseases, impacting gait and gait-related functional activities in ambulant children affected by these rare and often degenerative diseases. Gait speed is an indicator of health and disability, yet gait is a complex, multi-faceted activity. Using the International Classification of Function, Health and Disability (ICF) model, assessment of gait and functional ambulation should consider the impairments, activity limitations and participation restrictions due to disease, and factors related to the environment and the individual person.

**Methods:**

This narrative review involved a literature search of databases including Medline, Embase and Pubmed from 1946 to October 2019. Inclusion criteria included assessments of gait, endurance and ambulatory function in paediatric (0–18 years) neuromuscular diseases.

**Results:**

Fifty-two papers were identified reporting assessments of gait speed, timed function, endurance and ambulatory capacity, gait-related balance and qualitative descriptive assessments of gait function and effect of disease on gait and gait-related activities. Gait speed is an indicator of disability and children with neuromuscular disease walk slower than typically developing peers. Increasing disease severity and age were associated with slower walking in children with Duchenne muscular dystrophy and Charcot-Marie-Tooth disease. The six-minute walk test is used widely as a test of endurance and ambulatory capacity; six-minute walk distance was substantially reduced across all paediatric neuromuscular diseases. Endurance and ambulatory capacity was more limited in children with spinal muscular atrophy type 3, congenital muscular dystrophy and older boys with Duchenne muscular dystrophy. Only a few papers considered normalisation of gait parameters accounting for the effect on gait of height in heterogeneous groups of children and linear growth in longitudinal studies. Balance related to gait was considered in five papers, mainly in children with Charcot-Marie-Tooth disease. There was limited investigation of factors including distance requirements and terrain in children’s typical environments and personal factors related to self-perception of disease effect on gait and gait-related function.

**Conclusion:**

Assessments of gait and functional ambulation are important considerations in documenting disease progression and treatment efficacy in the clinical setting; and in clinical trials of disease-modifying agents and physiotherapeutic interventions in paediatric neuromuscular diseases. There is a need for expert consensus on core gait and functional ambulation assessments for use in clinical and research settings.

## Background

Neuromuscular diseases (NMD) include disorders affecting the anterior horn cells, peripheral nerves, neuromuscular junction and muscles, are often progressive and exhibit a wide range of impairment and disability in affected individuals. Relatively rare, in children these diseases include spinal muscular atrophy (SMA; 1 in 10–11,000) [1], Charcot-Marie-Tooth disease (CMT; 1 in 2.5–10,000) [2] and Duchenne muscular dystrophy (DMD; 1 in 3.5–10,0000) [3]. Gait and functional ambulation are important markers of disease and disability in paediatric NMD. It has been proposed that gait velocity or self-selected walking speed is the 6th vital sign of human function and is a predictor of overall health and disability [4, 5]. Yet, human gait is a complex activity and gait speed alone does not reflect all contributing factors and influences on gait [4, 5]. Functional ambulation relates gait function and speed to mobility-related activities of daily living in a person’s own environment [6]. Thus, the assessment of gait and functional ambulation in paediatric NMD must take in to consideration the many constructs that contribute to these complex tasks, including individual impairments, the task required, and the environment in which these are performed.

Weakness is the primary impairment in NMD, often presenting in childhood and adversely affecting gait and functional ambulation. Additionally, muscle tightness, contracture, musculoskeletal deformities, poor standing balance and reduced endurance, all arising from weakness, affect functional ambulatory tasks such as standing and walking. Patterns of weakness differ depending on the NMD, some affecting more proximal muscles (e.g. DMD) and others more distal muscles (e.g. CMT). For the majority of NMD of childhood, disease is progressive leading to increasing weakness and disability across the lifespan. The degree of disability in childhood NMD is variable ranging from children who are extremely weak and unable to sit (e.g. SMA type 1) to those with milder weakness who remain ambulant, albeit with gait difficulties including foot drop, poor balance and reduced gait speed (e.g. CMT, SMA type 3 and ambulant boys with DMD).

**Fig. 1:**
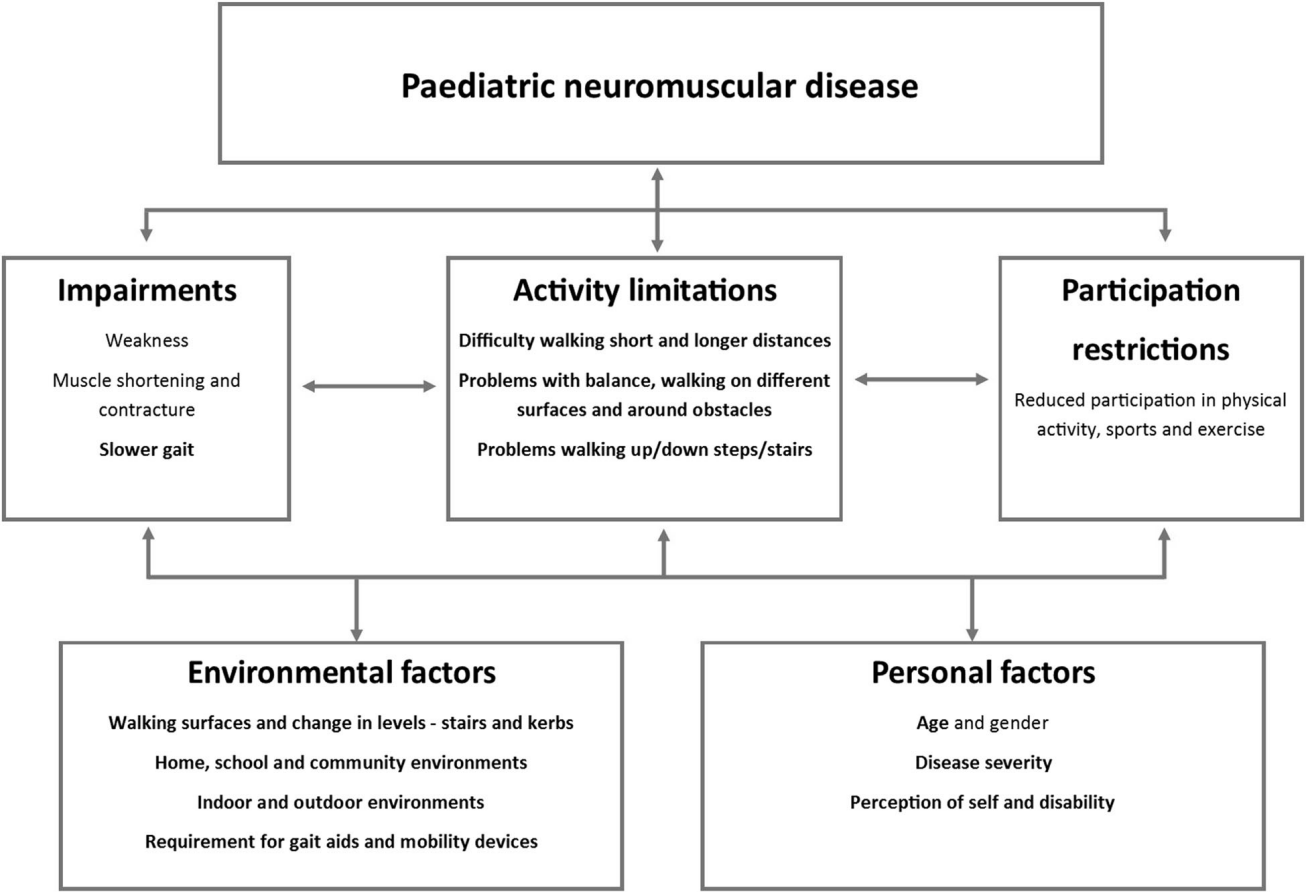
Paediatric neuromuscular disease as it relates to the ICF – factors relating to gait and functional ambulation (bold)

Gait dysfunction has important implications for function in everyday life. Ambulant children with NMD, commonly report problems with walking, poor balance, frequent trips and falls, increased fatigue and difficulty keeping up with peers [7–11]. In this context, it is useful to consider the assessment of gait and functional ambulation in ambulant children with NMD in terms of the International Classification of Function, Health and Disability (ICF) [12] (Fig. 1). Gait speed is an indicator of impairment. However, a clinically meaningful assessment also needs to consider limitations to the child’s activities, restrictions to participation, environmental and intrinsic personal factors. Activity limitations may include difficulty walking longer distances; problems with balance may affect steadiness and safety when walking; and ambulatory performance may be affected by changes in environmental conditions, for example walking over uneven ground or up and down steps. The use of gait or mobility aids will also affect function and low walking confidence or fear of falling may restrict participation opportunities. Assessments of functional ambulation need to take into account all of these factors. There is no recognised single assessment tool of functional ambulation that encompasses all factors. However, there are several clinical assessments that collectively illustrate the effect of disease and disability on gait and function in children with NMD. The aim of this paper is to review assessments of gait and functional ambulation in paediatric NMD.

## Method

This narrative review considered the literature relating to gait and functional ambulation in children and adolescents with neuromuscular disease. Literature was searched in Ovid Medline, Embase and PubMed from 1946 up to and including October 2019. Keywords and search terms included gait, walk*, ambul*, locomotion, functional ambul*, paed*, pediatr*, child*, adoles*, neuromuscular disease, Duchenne and Becker muscular dystrophy, Charcot-Marie-Tooth disease, peripheral neuropathy not diabetes, spinal muscular atrophy, myopathy, Pompe, myotonic dystrophy, collagen VI disorders, fascioscapulohumeral dystrophy or FSHD. Only full-text human studies in English were considered (see Supplementary material for example of search strategy). Additionally, hand searching of reference lists and conference abstracts was undertaken to ensure relevant publications were included. Exclusion criteria included studies of non-ambulant participants, primary studies of physical activity and activity monitoring that did not include assessments of gait or functional ambulation, and studies comprised primarily of adults (aged > 18 years).

## Results and discussion

### Studies included

Review of the literature resulted in 52 papers describing gait and functional ambulation in over eight neuromuscular diseases of childhood including CMT (15 papers), Becker and Duchenne muscular dystrophies (B/DMD) (21 papers), SMA (7 papers), congenital myotonic dystrophy (CMD) (2 papers), fascioscapulohumeral dystrophy (FSHD) (1 paper), Pompe disease (1 paper), collagen VI disorders (1 paper) and other mixed cohorts (4 papers) (Table 1). Two papers were systematic reviews of gait in CMT [18] and DMD [28]. Several papers described the same or similar cohorts, either cross-sectional and longitudinal studies [11, 16, 20, 21, 34, 35, 42] or a descriptive papers of a randomised controlled trial (RCT) [24, 25, 38, 39]. The median age of participants in the studies was 9.0 years and sample sizes ranged from 4 to 520. Some studies included adult participants and were included despite the lack of age-related sub-analysis [9, 10, 26, 36, 48–52, 56, 57, 60] or if there were no paediatric studies in a specific disease [55].

**Table 1:** Review papers including disease, author, study type, sample age and size, outcome measures and main findings

Disease	Study type Author	Sample size	Age years mean (SD)	Outcome measures	Gait speed m/s mean (SD)	6MWD m mean (SD)	10 m walk/run s mean (SD)	Other assessments
CMT	Cross-sectional, observational Estilow et al. 2019 [13]	CMT *n* = 520	10.9 (4.4)	Balance (BOT-2)				**↓ balance** Mean (SD) z-score − 3.25 (2.9)
	Cross-sectional, case-controlled observational Kennedy et al. 2019 [14]	CMT *n* = 50 TD *n* = 50	CMT 12.5 (3.9) TD 12.5 (3.9)	6MWT Balance (BOT-2)Walk-12		**↓ 6MWD** CMT 507.7 (137.3) TD 643.3 (75.6); *p* < 0.001 Norm to height CMT 341.9 (95.7) TD 429.4 (56.0); *p* < 0.001		**↓ balance** (BOT-2 /37) median (IQR) CMT 19 (9) TD 32 (3) *p* < 0.001 **Walk-12** mean score **24.7% (SD 19) 95% CI [19.3, 30.2]** *n* = 49
	Longitudinal observational Baptista et al. 2019 [15]	CMT *n* = 40 TD *n* = 49	CMT 11.45 (3.50) TD 10.62 (3.10)	10 m walk test (baseline)			**Slower 10 m walk** Median (25th; 75th) CMT 7.90 (6.67;8.92) TD 7.25 (5.60; 8.56); *p* ≤ 0.05	
	Cross-sectional, case-controlled observational Kennedy et al. 2018 [11] ^b^1	CMT *n* = 30 TD *n* = 30	CMT 11.5 (3.7) TD 11.5 (3.7)	Gait speed 6MWT 10 m walk/run (*n* = 26) Balance (BOT-2)	**Slower speed** CMT 1.19 (0.16) TD 1.32 (0.14); *p* < 0.001	**↓ 6MWD** CMT 557 (73) TD 615 (71); *p* < 0.001	**Slower 10 m walk/run** CMT 3.8 (1.0) TD 2.9 (0.3); *p* < 0.001	**↓ balance** (BOT-2 /37) Median (IQR) CMT 25.5 (4–34) TD 32 (26–35) *p* < 0.001
	Longitudinal cohort Kennedy et al. 2017 [16] ^b^1	CMT *n* = 27	Baseline 11.1 (3.7) 12 months 12.2 (3.7)	Gait speed non−/norm 6MWT non−/norm Balance (BOT-2) Functional mobility scale (FMS)	**Slower norm speed over 12 months** Baseline 1.18 (0.16) 12 months 1.15 (0.14); *p* = 0.22 Baseline 0.43 (0.07) 12 months 0.41 (0.05); *p* = 0.04	**↓ norm 6MWD over 12 months** Baseline 556 (73.7) 12 months 555 (80.2); *p* = 0.91 Baseline 720 (117) 12 months 690 (121); *p* = 0.006		**No change in balance over 12 months** (BOT-2 /37) Baseline 22.6 (9.4) 12 months 23.3 (8.7) *p* = 0.76 **FMS 78% reported reduced ambulatory function**
	Cross-sectional, observational cohort Cornett et al. (2016) [17]	CMT *n* = 520	10.9 (4.4)	6MWT		**↓ 6MWD in CMT genotypes** 6MWD z-scores reduced in CMT2A, CMT1B, CMT4C compared with CMT1A and CMTX1; *p* < 0.05 6MWD z-scores reduced in CMT2A and CMT4C compared with CMT1B; *p* < 0.05		
	Systematic review Kennedy et al. (2016) [18]	7 eligible studies	Mean 13 Range 2–52	Gait speed	**Slower speed** Range 0.50–1.25			
	Cross-sectional, observational Ounpuu et al. (2013) [19]	CMT *n* = 33 TD database	CMT 11.9 (3.6) TD 12.0 (3.0)	Gait speed	**Slower speed dependent on gait dysfunction** CMT toe walker 1.17 [0.21] CMT “typical” 1.11 [0.16]^a^ CMT foot drop 1.03 [0.21]^a^ TD control 1.27 [0.11]; *p* < 0.001 ^a^ compared with TD			
	Longitudinal Ferrarin et al. (2013) [20] ^b^2	CMT *n* = 16	13.2 (2.9)	Gait speed	**Non-significant changes in speed with large inter-subject variability**			
	Cross-sectional, case-controlled observational Ferrarin et al. (2012) [21] ^b^2	CMT *n* = 21 TD *n* = 18	CMT 11.9 (2.8) yo TD 11.0 (3.3) yo	Gait speed	**Slower norm speed** CMT1A foot drop and push-off deficit 69 [9] TD 77 [7]; *p* < 0.05 Norm to height			
	Cross-sectional observational Pagliano et al. (2011) [22]	*n* = 21	11.9 (2.8) range 6–17	Walk-12				**Walk-12** **Mean 12.4% (SD 9.5) Range 0–33**
	RCT Rose et al. (2010) [23] ^a^	CMT treatment group *n* = 15 CMT placebo group *n* = 15	CMT treatment 10 [4] yo CMT placebo 11 [3] yo	Gait speed	CMT treatment group 0.50 [0.10] CMT placebo group 0.60 [0.50]			
	RCT Burns et al. (2009a) [24] ^b^3	*n* = 81 [*n* = 53 for gait data; *n* = 65 for 6MWT data]	8.3 (3.5)	Gait speed 6MWT	Baseline CMT treatment group 1.19 (0.16) CMT placebo group 1.24 (0.19)	Baseline CMT treatment group 519 (86) CMT placebo group 521 (98)		
	Cross-sectional, observational Burns et al. (2009b) [25] ^b^3	*n* = 81 [*n* = 53 for gait data; *n* = 65 for 6MWT data]	8.3 (3.5)	Gait speed 6MWT	CMT 2–6 yo 1.13 (0.25) CMT 7–11 yo 1.25 (0.12) CMT 12–16 yo 1.23 (0.14)	CMT 2–6 yo 494.3 (70.6) 385–640 CMT 7–11 yo 526.8 (83.1) 250–640 CMT 12–16 yo 518.7 (150.2) 110–710		
	Cross-sectional, observational Newman et al. (2007) [26] ^a^	*n* = 16 TD database *n* = 40	CMT 20.1 (13) TD 18.4 (8.5)	Gait speed	**Slower speed** CMT 1.12 (0.17) TD controls 1.31 (0.13); *p* < 0.001			
DMD	Longitudinal Fowler et al. (2018) [27]	*n* = 42	7.9 (2.9) Range 4.1–16.1	10 m walk/run test StepWatch Activity monitoring	Baseline speed 1.71 (0.68) m/s as measured by 10 m walk/run		Baseline 5.85 s derived from reported gait speed	**Significant ↓ average strides per day with ↑ age**; per age group average strides per day ↑ for 4-7yo, plateau for 8-10yo and ↓ >10yo
	Systematic review of gait Goudriaan et al. (2018) [28]	9 eligible studies		Gait speed calculated from 6 studies	**Slower speed compared to TD** Standardised mean difference (effect size) ranging from 1.26 to 3.20			
	RCT Victor et al. (2017) [29]	*n* = 331	9.6 (1.92)	6MWT 10 m walk/run		Baseline 329.6 (55.47)	Baseline 6.8 (1.97)	
	Cross-sectional observational case controlled Alfano et al. (2017) [30]	DMD *n* = 72 TD *n* = 599	DMD range 4–12 TD range 4–14	100 m timed test				**Slower timed 100 m across all ages compared to TD;** *p* < 0.01 **DMD times improve up to 6 years and then decline from 7 years**
	Cross-sectional, observational case controlled Ropars et al. (2016) [31]	DMD *n* = 16 TD *n* = 15	DMD 8.67 (2.04) TD 9.39 (2.21)	Gait speed	**Slower speed compared to TD** DMD 0.78 (0.18) TD 1.21 (0.13); *p* < 0.001			
	Cross-sectional observational case-controlled Davidson et al. (2015) [32]	DMD *n* = 16 TD *n* = 13	DMD 9.0 (2.1) TD 9.0 (2.4)	6MWT StepWatch Activity monitoring		**↓ 6MWD compared to TD** DMD 387 (86) TD 598 (63); *p* < 0.005		**↓ steps and high activity time and ↑ inactive time compared to TD** Steps DMD 5138 (2500) TD 7239 (2621); *p* = 0.044 High activity time DMD 25 (17) TD 53 (34) min *p* = 0.018 Inactive time DMD 1103 (134) min TD 1016 [33] min; *p* = 0.036
	Longitudinal observational Pane et al. (2014) [34] ^b^4	*n* = 96	Baseline 8.3 (2.3)	6MWT		**↓ 6MWD 3 years** − 15.8 (77.3) at 12 months, − 58.9 (125.7) at 24 months and − 104.22 (146.2) at 36 months		
	Longitudinal observational Mazzone et al. (2013) [35] ^b^4	*n* = 113	8.2 4.1–17.0	6MWT		**↓ 6MWD 2 years** **−** 22.7 (SD 81.0) 1st year − 64.7 (SD 123.1) 2nd year		
	Longitudinal observational Henricson et al. (2013a) [36]	*n* = 340	Baseline range 2–28	10 m walk/run			**Slower 10 m walk/run with ↑ age** No participant aged > 18 years able to walk	
	Longitudinal observational, case-controlled Henricson et al. (2013b) [37]	DMD *n* = 24 TD *n* = 36 1 year DMD *n* = 13 TD *n* = 18	DMD 7.9 (2.3) TD 8.7 (2.6)	6MWT 10 m walk/run		**Baseline ↓ 6MWD** DMD 369.5 (79.3) TD 613.3 (73.6) *p* < 0.001 **MCID 26.4 m** **1 year change DMD > MCID** DMD − 53.67 (SE 25.96) *p* = 0.027 TD 16.5 (SE 11.46)	**Baseline ↓time** DMD 1.68 (0.56) TD 3.32 (0.36) *p* < 0.001 **MCID 0.19 s** **1 year change DMD > MCID** DMD − 0.25 (SE 0.68) *p* = 0.007 TD 0.33 (SE 0.07) *p* < 0.001	
	Observational study McDonald et al. (2013a) [38]	*n* = 174	8.5 (2.6)	6MWT 10 m walk/run		**358 (95)** **MCID 28.5–31.7 m**	**7.4 (4.3)** **MCID 2.3–1.4 s**	
	Longitudinal observational study McDonald et al. (2013b) [39]	*n* = 57	8.3 (2.33)	6MWT 10 m walk/run		**↓ 6MWD 48 weeks** Baseline 361.1 (87.5) Week 48,317.4 (152.3)	**Slower 10 m walk/run with ↑ age** Baseline < 7yo 4.8 (0.86) > 7yo 7.1 (2.80)	
	Longitudinal observational study Bello et al. (2012) [40]	*n* = 80 2 genotypes Group 1 = 57 Group 2 = 23	8.3 (2.7)	6MWT		**↓ 6MWD 12 months, associated with age** (*r* = − 0.38 *p* = 0.013), **baseline 6MWD** (*r* = 0.73, *p* < 0.001**) and genotype** (*p* = 0.029) Baseline Gp 1368 (86) Gp 2387 (67) 12 months Gp 1360 (98) Gp 2343 (124)		
	Cross-sectional, observational case controlled Doglio et al. (2011) [41]	DMD *n* = 15 TD *n* = 9	DMD 6.1 (0.7) TD 7.5 (1.2)	Gait speed 10 m walk	**Speed in younger boys with DMD does not differ to TD** DMD 1.06 (0.17) TD 1.07 (0.18) NS		**Slower 10 m walk** DMD 4.4 (3.2) TD 3.4 (0.6) *p* < 0.05	
	Longitudinal observational study Mazzone et al. (2011) [42] ^b^4	*n* = 106 (*n* = 100 10 m walk/run)	Baseline 8.3 (2.3)	10 m walk/run 6MWT		**↓ 6MWD 12 months, > in older boys** ≤ 7 yo − 7.8 (63.9) > 7 yo − 42.3 (73.9) All − 25.8 (74.3); *p* = 0.01 (≤7yo vs > 7yo)	**Slower 10 m walk/run in older boys** ≤ 7 yo 0.3 (3.1) > 7 yo 1.3 (3.5) All 1.0 (3.4) *p* = 0.11 ≤ 7yo vs >7yo	
	Cross-sectional observational study Mazzone et al. (2010) [43]	*n* = 112	8.18 (2.3)	10 m walk/run 6MWT		Range 127–560.6	Range 3–15	
	Cross-sectional observational case controlled McDonald et al. (2010) [44]	DMD *n* = 21 TD *n* = 34	Median [range] DMD 8 [5–12] TD 9 [4–12]	6MWT		**↓ 6MWD compared to TD** DMD 366 (83) TD 621 (68); *p* < 0.001		
	Cross-sectional observational case controlled Gaudreault et al. (2010) [8]	DMD *n* = 11 TD *n* = 14	DMD 9.2 (2.6) TD 9.7 (1.9)	Gait speed	**Slower speed** DMD 0.62 (0.12) TD 1.02 (0.13) *p* < 0.001			
	Cross-sectional, observational case controlled D’Angelo et al. (2009) [45]	DMD *n* = 21 TD *n* = 10	DMD 7.0 (2.4) TD 7.4 (1.2)	Gait speed	**Trend to slower norm speed** DMD 0.81 (0.14) TD 0.90 (0.13) norm to height; NS			
	RCT Skura et al. (2008) [46]	*n* = 15	Baseline 8.4 (1.46)	30 ft timed walk/run test			Baseline 30 ft timed run 5.6 (1.3)	
	RCT Beenakker et al. (2005) [47]	*n* = 16	Baseline 6.25 (0.93) Range 5–8	9 m run			Baseline speed 1.78 m/s = 5.06 s over 9 m	
SMA	Pilot study Bartels et al. (2019) [48]	*n* = 4	26.2; range 10–37	Endurance shuttle walk test (ESWT)				**ESWT feasible measure of fatigability during walking in SMA3**
	Longitudinal, observational Montes et al. (2018) [10]	*n* = 73	13.5 (12.4) range 2.6–49.1	6MWT		Baseline SMA3a 257.1 (107.3) SMA3b 390.2 (144.0) **Mean rate of change − 7.8 m/year**; *p* = 0.009 **Age affects rate of change** < 6 yrs.: 9.8 m/ year; 6–10 yrs.: − 7.9 m/year; 11–19 yrs.: − 20.8 m/year; > 20 yres: − 9.7 m/year; *p* = 0.005		
	Cross-sectional, observational Montes et al. (2014) [49]	*n* = 10	31.2; range, 9–49	6MWT		273.4 (45.7); range, 53–492		
	Dunaway et al. (2014) [50]	*n* = 15	28.7; range 10–49	10 m walk/run 6MWT Timed up and go (TUG)		362.13 (29.22)	7.44 (0.84)	TUG 12.97 (2.49) s; **Moderate to good correlation between TUG and 10 m walk/run** (*r* = 0.691; *p* = 0.009) **and 6MWT** (*r* = − 0.514; *p* < 0.05)
	Longitudinal observational study Mazzone et al. (2013) [51]^a^	*n* = 38	14.07 (12.43) 3.4–49.3	6MWT		**No change in 6MWD 12 months** Baseline 294.91 (127) 12 months 293.4 (141)		
	Cross-sectional, case controlled Montes et al. (2011) [9]	SMA3 *n* = 9 TD *n* = 9	22 4–49 years	6MWT		**↓ 6MWD** SMA3 343 m (range 267–449) TD 601 m (range 490–733)		
	Cross-sectional observational Montes et al. (2010) [52]	*n* = 18	15.3(13.3)	6MWT 10 m walk/run		288.9 (161.9) **Strong correlation between 6MWT and 10 m walk/run** (*r* = − 0.87; *p* < 0.0001)	Median (25th, 75th %) 8.4 (5.4,10.7)	
CMD	Cross-sectional observational Hayes et al. (2018) [53]	*n* = 25	7.76 (3.02) 3.25–13.22	10 m self-selected walk speed 6MWT StepWatch activity monitoring	0.97 (0.26) 0.52–1.36	325.23 (109.85) 150–604		**↓ physical activity** Time inactive, % 80.85 (9.15) Low steps, % 33.19 (13.11) Medium steps, % 51.15 (9.80) High steps, % 16.01 (10.27)
	Cross-sectional observational case-controlled Johnson et al. (2016) [54]	CMD *n* = 41 (6MWT *n* = 33) TD *n* = 29	CMD 6.8 (3.3) TD 9.1 (3.1)	6MWT		**↓ 6MWD** CMD 258.3 m (SD 176) TD 568.3 m (SD 73.2); *p* < 0.001		
FSHD	Observational test-retest reliability study Eichinger et al. (2017) [55]^a^	*n* = 86	49.1 (15.2) 18–84	6MWT		404.3 (123.9); Reliability ICC = 0.99 (*n* = 25) **Minimal detectable change (MDC95) 34.3**		
Late onset Pompe	Observational cross-sectional McIntosh et al. (2015) [56]^a^	*n* = 22 Gender age-matched reference data	48.6 (range 13–72)	Gait speed 6MWT 10 m fast walk test	1.02 (0.30)	**Variable performance ranging from 39.4 to 110% predicted**	10 m fast walk 1.41 (0.42) m/s = 7.09 s	
Collagen VI	Longitudinal observational Meilleur et al. (2015) [57]^a^	*n* = 32 (*n* = 11 for 10 m walk/run and 6MWT)	Range 4.8–21.2	6MWT 10 m walk/run		338.27 (126.65) 144–600	10 m walk 8.6 (3.5) 4.0–15.8	
NMD (mixed)	Cross-sectional observational Witherspoon et al. (2019) [58] ^c^	*n* = 77	10.1 ± 2.93	6MWT 2MWT		442.1 (121.6)		2MWD = 149.8 (40.3) m **Strong correlation 2MWD and 6MWD** ***r*** **= 0.90,** ***p*** **< 0.01**
	Cross-sectional observational Kaya et al. (2015) [59]	*n* = 40 DMD = 20 PN ^d^ = 20	DMD 9.05 (3.1) PN 12.95 (3.3)	6MWT TUG		DMD 349.70 (77.18) PN 358.85 (75.07) NS		TUG DMD 7.79 (1.54) s PN 10.13 (2.63) s *p* < 0.01
	Cross-sectional observational Montes et al. (2013) [60] ^a e^	*n* = 114	21.3 (range 4–64)	6MWT (% predicted of normative reference data)		Mean 61.9% DMD/BMD 65.1% SMA 52.0% myasthenia gravis 66.3% GLUT1 deficiency/mitochondrial disorders 63.9%		
	Cross-sectional observational Holtebekk (2013) [61] ^f^	*n* = 17	Median (IQR) 14.2 (3.6)	6MWT Activity monitoring SenseWear Armband		Median (IQR) 485 (131)		Activity monitor Moderate physical activity Median (IQR) 2.4 (1.9) hours/weekday 1.1 (3.3) hours/weekend day

Mean and standard deviation (SD) unless otherwise stated; IQR = inter-quartile rank; Main findings in bold

Abbreviations: *6MWT* six-minute walk test, *6MWD* six-minute walk distance, *BMD* Becker muscular dystrophy, *BOT-2* Bruininks-Oseretsky Test, 2nd Edition, *CMT* Charcot-Marie-Tooth disease, *CMD* Congenital myotonic dystrophy, *DMD* Duchenne muscular dystrophy, *ESWT* Endurance shuttle walk test, *FMS* Functional Mobility Scale, *FSHD* fascioscapulohumeral dystrophy, *LGMD* limb girdle muscular dystrophy, *NMD* neuromuscular disease, *norm* norm, *PN* peripheral neuropathies, *SMA* spinal muscular atrophy

^a^Nelson - included adult participants with no sub analysis; McIntosh – 2 of 22 subjects aged ≤18 years, no sub-analysis; Mazzone - included adult participants with no sub analysis; Eichinger – one 18-year-old participant, all others adults with no sub-analysis; Meilleur - included adult participants with no sub analysis; Montes - included adult participants with no sub analysis

^b^ same data sets

^c^Collagen VI-related dystrophy (COL6-RD), laminin alpha 2-related dystrophy (LAMA2-RD), limb-girdle muscular dystrophy (LGMD), and RYR1-related myopathies (RYR1-RM), and other

^d^PN = peripheral neuropathies including CMT/hereditary motor and sensory neuropathy (HMSN), motor neuropathy (MN) and polyneuropathy (PNP)

^e^Spinal muscular atrophy (*n* = 23), Duchenne/Becker muscular dystrophy (*n* = 29), myasthenia gravis (*n* = 12), or an energy failure syndrome (glucose transporter protein type 1 [GLUT1] deficiency/mitochondrial disorders) (*n* = 50)

^f^CMT *n* = 4, Congenital myopathy *n* = 2, LGMD 2I *n* = 8, BMD *n* = 1, Unspecified *n* = 2

### Assessments of gait and functional ambulation

Several assessments of gait and functional ambulation in paediatric NMD were identified and discussed in the literature. These included timed function tests, for example the 10 m walk and/or run test [62, 63], the six-minute walk test (6MWT) [64] and 100 m timed test [30]. Tests of dynamic balance in walking, including the balance subset of the Bruininks-Oseretsky Test, 2nd edition (BOT-2) [65] and the timed up and go (TUG) [66], were considered due to the impact of balance on gait and function. If studies of gait or functional ambulation included activity monitoring, these were reported as an adjunct to specific assessments of gait, however we did not specifically search for studies of activity monitoring alone. We also included descriptive scales or questionnaires that characterised gait-related function, including the Functional Mobility Scale (FMS) [67] and the Walk-12 scale [68].

#### Gait speed

For this review we focused on gait speed in terms of its clinical utility as an indicator of health and disability [4, 5]. Assessment of gait speed was diverse with a range of reported methodologies including 3-D gait analysis, electronic walkway and timed distance. Typically, gait is assessed at natural self-selected steady walking pace, however some studies reported gait speed as calculated from the 10 m walk/run, usually a fast walk or run test [27, 56]. Gait speed was described most often in studies of children with CMT (ten of fifteen papers), a reflection of the use of clinical gait analysis in preparation for orthopaedic surgery for the management of foot deformities common in CMT [11, 16, 18–21, 23–26]. Children with CMT walked more slowly than their typically developing peers and a decline in gait speed over time was evident when growth was accounted for by normalising gait speed to height or leg length [16]. Greater disability in children with CMT was reflected in slower gait speed in more severely affected children [19].

Younger boys (< 7 years) with DMD did not walk significantly slower than their typically developing peers [41, 45]. However, boys older than 8 years with DMD were significantly slower than their peers, an indicator of the relentless and degenerative muscle disease [8, 31]. The study by Doglio et al. (2011) was the only study to normalise gait speed to height when comparing boys with DMD to TD controls. Normalising gait parameters is an important consideration for boys with DMD who are typically treated with corticosteroids, of which growth retardation is a known side-effect. Therefore, steroid-treated boys with DMD tend to be smaller than their age-matched, steroid naïve and non-affected peers [69]. Normalisation to height and/or leg length is an important to factor when reporting gait speed in paediatric populations [70, 71].

Gait speed was reported in two other studies in children with CMD [53] and a mixed aged study of children and adults with late-onset Pompe disease [56]. Whilst neither study included unaffected controls, the reported gait speed for both cohorts was considerably slower than reported normative reference data [72].

#### Six-minute walk test

The 6MWT is a valid and reliable standardised test of physical endurance in boys with DMD [38]. A test of ambulatory capacity, the 6MWT measures distance walked in six minutes. Developed from the American Thoracic Society and FDA-approved 6MWT [64], the test has been modified for children with NMD with the addition of a safety chaser and standardised verbal encouragement [44]. The utility of the 6MWT in paediatric NMD clinical research is evident with over half of the studies included in this review reporting its use [9–11, 14, 16, 17, 24, 25, 29, 32–35, 37–40, 42–44, 50–61] (Table 1). Typical walkway distances were reported as 25 m however some studies used walkways as short as 10 m [24, 25]. This may reduce distance walked due to more frequent turns resulting in greater time spent decelerating and less time at a fast walking pace [73].

Six-minute walk distance (6MWD) is reduced in children and adolescents with NMD when compared to typically developing controls or normative reference data. In boys with DMD and children with SMA type 3 and CMT, 6MWD declines with increasing age, reflecting disease progression and increasing disability [10, 16, 34, 35, 37, 39, 40]. Normalisation of 6MWD to height or leg length, accounts for linear growth across age groups and in longitudinal studies, and is an important factor when determining the effect of disease on function [16]. Burns and colleagues (2009), demonstrated a trend to shorter 6MWD in older children [25]. In a further study over 12 months, normalisation of 6MWD accounting for growth revealed a decline in ambulatory capacity in children with CMT [16]. Genotype also affects ambulatory capacity in the 6MWT; children with milder subtypes of CMT in a large study of 520 participants, walked further than children with more severe subtypes [17]. Deterioration in ambulatory capacity over time was also affected by genotype in 80 boys with DMD [40]. Several studies have reported rates of change, either minimally clinically important difference (MCID) or minimal detectable change (MDC) for 6MWD in different NMD including DMD (MCID 26.4–31.7 m; − 53.67 change over 12 months) [37, 38], SMA (− 7.9 to − 9.7 m over 12 months) [10] and FSHD (MDC95 34.3 m) [55]. Measures of rate of change, MCID or MCD are useful for clinicians when comparing the ambulatory function of the children in their own clinical practice.

#### Timed function tests – 10 m walk/run

The timed 10 m walk/run is widely used to assess function in NMD and is a sensitive measure of disease progression in ambulant boys with DMD [62, 74, 75]. In clinical practice, the timed 10 m walk/run is conducted as a fast walk or run dependent on the abilities of the child. However, in the literature there were differences noted between whether the test was conducted as a timed walk [15, 41, 56, 57] or run, and earlier studies conducted the test over 30 ft or 9 m [46, 47]. Timed 10 m walk/run was most often reported in boys with DMD [27, 29, 36–39, 41–43, 46, 47] reflecting its common use as a predictor of disease in DMD and less frequently in studies of CMT, SMA, Pompe and Collagen VI [11, 15, 50, 52, 56, 57]. Across all NMDs, 10 m walk/run times were slower compared to controls or reported normative reference ranges. Disease progression in boys with DMD was demonstrated with several studies reporting slower speed over 10 m in boys older than 7 years [36, 39, 42].

#### Other assessments

Assessments of balance, endurance and alternative distance and timed tests (100 m timed walk and two-minute walk test) were reported less frequently. The balance subset of the Bruininks-Oseretsky Test of Motor Proficiency, 2nd Ed (BOT-2, NCS Pearson, Upper Saddle River, NJ, USA) is a ten-item standardised test of balance in standing and walking [65]. It is widely used in paediatric CMT having been incorporated into the disease-specific CMT Pediatric Scale (CMTPedS) [76]. Balance in children with CMT was significantly reduced when compared to age- and gender-matched controls and normative reference data [11, 13, 14]. However, over 12 months’, balance did not significantly deteriorate, indicative of the relatively slow progression of neuropathy in children with CMT [16]. Reduced balance (BOT-2) was associated with wider base of support and greater step-to-step variability in the gait of children with CMT [11].

The TUG was reported in two studies and is a valid, reliable and responsive measure of gait-related balance in children with physical disabilities [50, 59, 77, 78]. It measures the time taken to stand from a seated position, walk 3 m, turn around and return to a seated position. Dunaway and colleagues (2014) demonstrated an association between the TUG, slower 10 m walk/run time and shorter 6MWD in children with SMA type 3 [50]. In a further study comparing older children with peripheral neuropathies (PN) of mixed origin to younger boys with DMD, TUG times were longer in the children with mixed PN [59]. The authors attributed the difference between groups to the effects of distal versus proximal weakness patterns, however it may have also been due to the PN group being older and therefore likely to be more affected by their disease. Both the BOT-2 and the TUG are useful assessments of gait-related balance and inform clinical interpretation of the effects of NMD on gait and function.

Alternatives to the 6MWT were reported in two studies. Alfano and colleagues (2017) reported the development and validation of a 100 m timed test in boys with DMD as an assessment of functional ambulatory performance. Their study included the establishment of typically developing normative reference data [30]. Findings from this study indicated that performance on the 100 m timed test improved up to the age of 7 years before plateauing and declining with increasing age [30]. This finding is similar to the 10 m walk/run and the 6MWT in boys with DMD and is reflective of disease progression [36, 39, 40, 42]. A second study in children with mixed NMD, reported a two-minute walk distance (2MWD) derived from the two-minute mark of the standard 6MWT, and found a strong correlation between 2MWD and 6MWD [58]. Both the 100 m timed test and the 2MWT may offer realistic alternatives to the longer 6MWT, especially in children with behavioural and attentional problems who may find the time and testing constraints of six minutes challenging.

The 6MWT and more recently the 100 m timed test and 2MWT, are all described as tests of physical endurance in paediatric NMD and as surrogate measures of physical fatigue in DMD, SMA and CMT [9, 25, 44]. Bartels and colleagues (2019) have recently described the concept of fatigability in SMA, that is, “*the inability to perform prolonged repetitive tasks during activities of daily life”*, and have developed a group of endurance tests to assess fatigability [48]. The Endurance Shuttle Walk Test is a feasible test of endurance in ambulant people with SMA type 3. An externally paced walking task, the objective is to cover 10 m before each beep. The time between repeated beeps decreases, requiring an incremental increase in walking speed. This pilot study is the first report of the Endurance Shuttle Walk test and further studies are required to determine its validity, responsiveness and clinical utility in SMA and paediatric NMD.

The advent of wireless wearable activity monitors allows remote measurement of gait-related physical activity in children with NMD. Several studies have included wearable devices to measure steps and physical activity in DMD, CMD and mixed NMD [27, 32, 53, 61]. Davidson and colleagues (2015) found that boys with DMD have reduced daily step counts and reduced high activity time, with correspondingly greater inactivity compared to typically developing peers [32]. In a longitudinal study, Fowler and colleagues (2018) described physical activity levels in boys with DMD following a similar trajectory to timed tests and the 6MWT [27]. Daily step counts rose up to the age of 8 years, before plateauing and declining from the age of 10 years [27]. Children with NMD are largely inactive, spending up to 80% of their awake time in sedentary activities and on average only 2 h a day at a moderate intensity of activity [53, 61]. Whilst we assume neuromuscular weakness contributes to reduced physical activity, it is quite possible that reduced physical activity contributes further to increasing weakness. Lack of physical activity, together with disease-related weakness, is likely to impact gait and function in children with NMD.

#### Descriptive measures of gait

Together with quantitative measures of gait and function, descriptive scales and questionnaires are used to characterise gait and functional ambulation in paediatric NMD. Two such measures reported in this review are the Functional Mobility Scale (FMS) [67] and the Walk-12 [68] which have not been widely utilised in paediatric NMD to date.

The Functional Mobility Scale (FMS) is a clinical tool used to classify typical mobility over three distance categories – 5, 50 and 500 m in children with gait dysfunction [67]. These distances are commensurate with walking in the home (5 m), between classrooms in the school environment (50 m) and distances required in general community environments (500 m). The scale considers the level of assistance the child requires and a grading from 1 to 6 is applied; where 1 indicates the use of a wheelchair and 6 indicates fully independent walking on all surfaces and terrains, including stairs without use of a rail. One study has described functional mobility in children with CMT [16]. In this study 78% of the children reported reduced ambulatory function, across one or all distance/environment categories (score < 6) describing the functional impact of disease. Over 12 months there was little change in FMS scores, in keeping with the slow progression of CMT.

The Walk-12 is a self-reported questionnaire of perceived impact of disease on gait and gait related activities, such as running, using stairs and balance, modified and validated in adults with peripheral neuropathies [68, 79]. The tool is scored from 0 to 100%, where 0% indicates no impact and 100% indicates a high impact of disease on gait and related activities. In two studies, Walk-12 scores ranged from 12 to 25%, indicating that children with CMT perceived only mild impact of disease on gait and gait-related function [14, 22]. Interestingly, further examination of answers to individual questions indicated that CMT affected their ability to run (43%), ascend or descend stairs (41%), the speed with which they could walk (31%) and made it more effortful to walk (31%) [14]. Additionally, over 40% of the children reported moderate to severe limitations to their ability to walk longer distances in their everyday environments.

### Clinical implications and future directions

Gait performance and functional ambulation are biomarkers of disease severity in paediatric NMD, providing a measure of disability. Assessment of gait and functional ambulation are important outcome measures in the toolbox of assessments for clinical research trials and in the clinical setting. With the advent of disease-modifying pharmacological treatments and uptake of physiotherapeutic exercise, younger people, including children with NMD are likely to benefit most from treatments. Therefore, it is important to utilise functional measures specific to children and adolescents to monitor disease progression and treatment efficacy.

There remains a need for further research and development of functional gait outcome measures for paediatric NMD. Limitations including the heterogeneity of the study populations and differences in assessment protocols precluded meta-analysis of these studies. ​As evidence accumulates, a more analytical systematic review may provide additional insights. The disparity of gait assessments in paediatric neuromuscular diseases also suggests that a Delphi survey would be useful in establishing expert consensus on which measures to use. Factors such as the normalization of gait parameters is important when comparing gait across age ranges and longitudinally to account for growth in children and should be standardly applied both clinically and in research. Further development and publication of paediatric normative reference datasets and MCIDs will be a valuable resource for clinicians working in neuromuscular clinics. Qualitative characterisation of gait and functional ambulation with scales or questionnaires enables clinicians to gain an understanding of the effect of disease on the day-to-day lives of children with NMD, beyond the neuromuscular outpatient clinic. The self-reported scales of functional mobility and gait-related activities require validation in paediatric NMD populations to ensure that they provide meaningful information and enable greater uptake by clinicians. Further exploration and utilization of technology for remote and wireless monitoring and measurement of walking in the typical environments of children, including home, school and the community provide greater understanding of the effect of disease on function.

## Conclusion

This narrative review has highlighted clinical measures of gait ranging from gait speed to tests of ambulatory capacity, physical endurance and gait-related balance that can be conducted in clinical and research settings with relative ease. Consideration of environmental factors affecting function including distance requirements necessitated by school and community settings is important when understanding the effect of disease. An individual’s perception of the effect of disease on walking and everyday function are important considerations in the clinical setting. Person-centred characterization and assessment of gait dysfunction discussed in this narrative review provides a rich and holistic illustration of disability, and offers genuine outcome measures of potential therapeutic benefits on gait and functional ambulation in paediatric NMD. However, consensus is required amongst experts in paediatric neuromuscular disorders to establish a core set of gait and functional ambulatory assessments that can be used clinically and in research settings.

## Supplementary information

**Additional file 1.** Search strategy for narrative review “Walking and weakness in children: a narrative review of gait and functional ambulation in paediatric neuromuscular disease”.

## Data Availability

All articles cited in this review are accessible through article repositories and journal websites.
