# Supplementary material for: Walking and weakness in children: a narrative review of gait and functional ambulation in paediatric neuromuscular disease
Source: J Foot Ankle Res. 2020 Mar 2;13:10. doi: 10.1186/s13047-020-0378-2 (PMC7052968; doi:10.1186/s13047-020-0378-2)
Supplement: Supplementary file 1 — Additional file 1. Search strategy for narrative review “Walking and weakness in children: a narrative review of gait and functional ambulation in paediatric neuromuscular disease”. [file 13047_2020_378_MOESM1_ESM.docx]

| **Search strategy for narrative review “Walking and weakness in children: a review of gait and functional ambulation in paediatric neuromuscular disease”** |
| --- |
| 1. (gait or walk* or ambul* or locomotion).mp. [mp=title, abstract, original title, name of substance word, subject heading word, floating sub-heading word, keyword heading word, organism supplementary concept word, protocol supplementary concept word, rare disease supplementary concept word, unique identifier, synonyms] |
| 2. limit 1 to (english language and full text and humans) |
| 3. functional ambulation.mp. [mp=title, abstract, original title, name of substance word, subject heading word, floating sub-heading word, keyword heading word, organism supplementary concept word, protocol supplementary concept word, rare disease supplementary concept word, unique identifier, synonyms] |
| 4. limit 3 to (english language and full text and humans) |
| 5. 2 or 4 |
| 6. (paed* or pediatr* or child* or adoles*).mp. [mp=title, abstract, original title, name of substance word, subject heading word, floating sub-heading word, keyword heading word, organism supplementary concept word, protocol supplementary concept word, rare disease supplementary concept word, unique identifier, synonyms] |
| 7. limit 6 to (english language and full text and humans) |
| 8. neuromuscular disease.mp. [mp=title, abstract, original title, name of substance word, subject heading word, floating sub-heading word, keyword heading word, organism supplementary concept word, protocol supplementary concept word, rare disease supplementary concept word, unique identifier, synonyms] |
| 9. limit 8 to (english language and full text and humans) |
| 10. Duchenne muscular dystrophy.mp. [mp=title, abstract, original title, name of substance word, subject heading word, floating sub-heading word, keyword heading word, organism supplementary concept word, protocol supplementary concept word, rare disease supplementary concept word, unique identifier, synonyms] |
| 11. limit 10 to (english language and full text and humans) |
| 12. Becker muscular dystrophy.mp. [mp=title, abstract, original title, name of substance word, subject heading word, floating sub-heading word, keyword heading word, organism supplementary concept word, protocol supplementary concept word, rare disease supplementary concept word, unique identifier, synonyms] |
| 13. limit 12 to (full text and humans and latest update) |
| 14. ((Charcot-Marie-Tooth disease or peripheral neuropathy) not diabetes).mp. [mp=title, abstract, original title, name of substance word, subject heading word, floating sub-heading word, keyword heading word, organism supplementary concept word, protocol supplementary concept word, rare disease supplementary concept word, unique identifier, synonyms] |
| 15. limit 14 to (english language and full text and humans) |
| 16. spinal muscular atrophy.mp. [mp=title, abstract, original title, name of substance word, subject heading word, floating sub-heading word, keyword heading word, organism supplementary concept word, protocol supplementary concept word, rare disease supplementary concept word, unique identifier, synonyms] |
| 17. limit 16 to (english language and full text and humans) |
| 18. myopathy.mp. [mp=title, abstract, original title, name of substance word, subject heading word, floating sub-heading word, keyword heading word, organism supplementary concept word, protocol supplementary concept word, rare disease supplementary concept word, unique identifier, synonyms] |
| 19. limit 18 to (english language and full text and humans) |
| 20. Pompe.mp. [mp=title, abstract, original title, name of substance word, subject heading word, floating sub-heading word, keyword heading word, organism supplementary concept word, protocol supplementary concept word, rare disease supplementary concept word, unique identifier, synonyms] |
| 21. limit 20 to (english language and full text and humans) |
| 22. myotonic dystrophy.mp. [mp=title, abstract, original title, name of substance word, subject heading word, floating sub-heading word, keyword heading word, organism supplementary concept word, protocol supplementary concept word, rare disease supplementary concept word, unique identifier, synonyms] |
| 23. limit 22 to (english language and full text and humans) |
| 24. collagen VI disorders.mp. [mp=title, abstract, original title, name of substance word, subject heading word, floating sub-heading word, keyword heading word, organism supplementary concept word, protocol supplementary concept word, rare disease supplementary concept word, unique identifier, synonyms] |
| 25. limit 24 to (english language and full text and humans) |
| 26. (fascioscapulohumeral dystrophy or FSHD).mp. [mp=title, abstract, original title, name of substance word, subject heading word, floating sub-heading word, keyword heading word, organism supplementary concept word, protocol supplementary concept word, rare disease supplementary concept word, unique identifier, synonyms] |
| 27. limit 26 to (english language and full text and humans) |
| 28. 9 or 11 or 13 or 15 or 17 or 19 or 21 or 23 or 25 or 27 |
| 29. 5 and 7 and 28 |
